# Prognostic value of computed tomography radiomics features in patients with gastric neuroendocrine neoplasm

**DOI:** 10.3389/fonc.2023.1143291

**Published:** 2023-06-20

**Authors:** Zhi-hao Yang, Yi-jing Han, Ming Cheng, Rui Wang, Jing Li, Hui-ping Zhao, Jian-bo Gao

**Affiliations:** ^1^ Department of Radiology, The First Affiliated Hospital of Zhengzhou University, Zhengzhou, China; ^2^ Henan Key Laboratory of Image Diagnosis and Treatment for Digestive System Tumor, The First Affiliated Hospital of Zhengzhou University, Zhengzhou, China; ^3^ Department of Medical Information, The First Affiliated Hospital of Zhengzhou University, Zhengzhou, China; ^4^ Department of Radiology, Affiliated Tumor Hospital of Zhengzhou University, Zhengzhou, China; ^5^ Department of Radiology, Shanxi Provincial People’s Hospital, Xi’an, China

**Keywords:** gastric neuroendocrine neoplasm, tomography, x-ray computed, radiomics, prognosis

## Abstract

**Purpose:**

The present study aimed to investigate the clinical prognostic significance of radiomics signature (R-signature) in patients with gastric neuroendocrine neoplasm (GNEN).

**Methods and Materials:**

A retrospective study of 182 patients with GNEN who underwent dual-phase enhanced computed tomography (CT) scanning was conducted. LASSO-Cox regression analysis was used to screen the features and establish the arterial, venous and the arteriovenous phase combined R-signature, respectively. The association between the optimal R-signature with the best prognostic performance and overall survival (OS) was assessed in the training cohort and verified in the validation cohort. Univariate and multivariate Cox regression analysis were used to identify the significant factors of clinicopathological characteristics for OS. Furthermore, the performance of a combined radiomics-clinical nomogram integrating the R-signature and independent clinicopathological risk factors was evaluated.

**Results:**

The arteriovenous phase combined R-signature had the best performance in predicting OS, and its C-index value was better than the independent arterial and venous phase R-signature (0.803 vs 0.784 and 0.803 vs 0.756, P<0.001, respectively). The optimal R-signature was significantly associated with OS in the training cohort and validation cohort. GNEN patients could be successfully divided into high and low prognostic risk groups with radiomics score median. The combined radiomics-clinical nomogram combining this R-signature and independent clinicopathological risk factors (sex, age, treatment methods, T stage, N stage, M stage, tumor boundary, Ki67, CD56) exhibited significant prognostic superiority over clinical nomogram, R-signature alone, and traditional TNM staging system (C-index, 0.882 vs 0.861, 882 vs 0.803, and 0.882 vs 0.870 respectively, P<0.001). All calibration curves showed remarkable consistency between predicted and actual survival, and decision curve analysis verified the usefulness of the combined radiomics-clinical nomogram for clinical practice.

**Conclusions:**

The R-signature could be used to stratify patients with GNEN into high and low risk groups. Furthermore, the combined radiomics-clinical nomogram provided better predictive accuracy than other predictive models and might aid clinicians with therapeutic decision-making and patient counseling.

## Introduction

Gastric neuroendocrine neoplasms (GNEN) are a group of rare and heterogeneous neoplasms originating from gastric neuroendocrine cells, with the fifth highest rate in the entire digestive tract (7.2%) (1). In the past 40 years, with the improvement of the understanding of GNEN and the progress of pathological diagnosis technology, the diagnostic rate and incidence of GNEN have been increasing year by year. The increase of GNEN ranks the first among all the incidence sites of neuroendocrine tumors, and the overall survival rate of GNEN is the second worst, inferior to pulmonary neuroendocrine tumors (2).

In 2019, the latest version of the World Health Organization classification criteria classified neuroendocrine neoplasm into highly differentiated neuroendocrine tumors (NET) and poorly differentiated neuroendocrine carcinoma (NEC), as well as mixed neuroendocrine-nonneuroendocrine neoplasm (MiNEN) with both highly and poorly differentiated components (3). At present, there were problems of over-treatment and inconsistent treatment protocols for NET in clinical practice, and insufficient attention was paid to NEC and MiNEN due to its rarity and related studies were few. However, in fact, patients with NEC or MiNEN had a worse prognosis and were more likely to have distant recurrence than patients with gastric adenocarcinoma (4).

So far, the most commonly used prognostic assessment tools for GNEN are the American Joint Committee on Cancer (AJCC) and the European Society of Neuroendocrine Neoplasms (ENETS) TNM staging system (5). Several previous studies have shown a distinct biological behavior between NET and NEC, with the latter being more aggressive and having worse prognosis (6, 7), but the system based solely on anatomically relevant prognostic factors, does not fully reflect tumor heterogeneity in predicting the long-term prognosis of NEN (8–11). In addition, in current clinical practice, two different protocols are used for GNEN staging, gastric neuroendocrine tumor (GNET) follows AJCC’s 8th edition of gastroenteropancreatic neuroendocrine tumor staging, while gastric neuroendocrine carcinoma (GNEC) follows AJCC’s 8th edition of gastric cancer. The two different protocols are not convenient for clinical use (12). Therefore, the current AJCC and ENETS TNM staging system still needs to be further improved, and it is urgent to find a novel and reliable biomarker to ensure a more accurate and convenient prediction of the prognosis of GNEN patients (13).

Conventional CT imaging can be used to predict the prognosis of patients with gastric cancer and GNEN (14, 15). However, the prognostic utility of conventional medical imaging is inherently limited by mutual accuracy and reproducibility. Advances in medical imaging technology and analytical methods have led to the development of radiomics, which is dedicated to transforming these medical images into high-dimensional, mineable data that can be used to objectively and quantitatively describe tumor phenotypes in a robust and reproducible manner (16). Radiomics enables non-invasive analysis of tumor heterogeneity and has been successfully applied to predict metastasis, recurrence and other clinical outcomes of renal, lung, breast and colorectal cancers (17–21). However, radiomics-based research for prediction prognosis of GNEN patients are still lacking. In this study, we develop and validate a radiomics signature (R-signature) for the prediction of OS in GNEN patients and subsequently to determine whether a novel nomogram combining R-signature and independent clinicopathological risk factors could provide more accurate prognostic prediction in such patients.

## Materials and methods

### Patients

**Figure 1:**
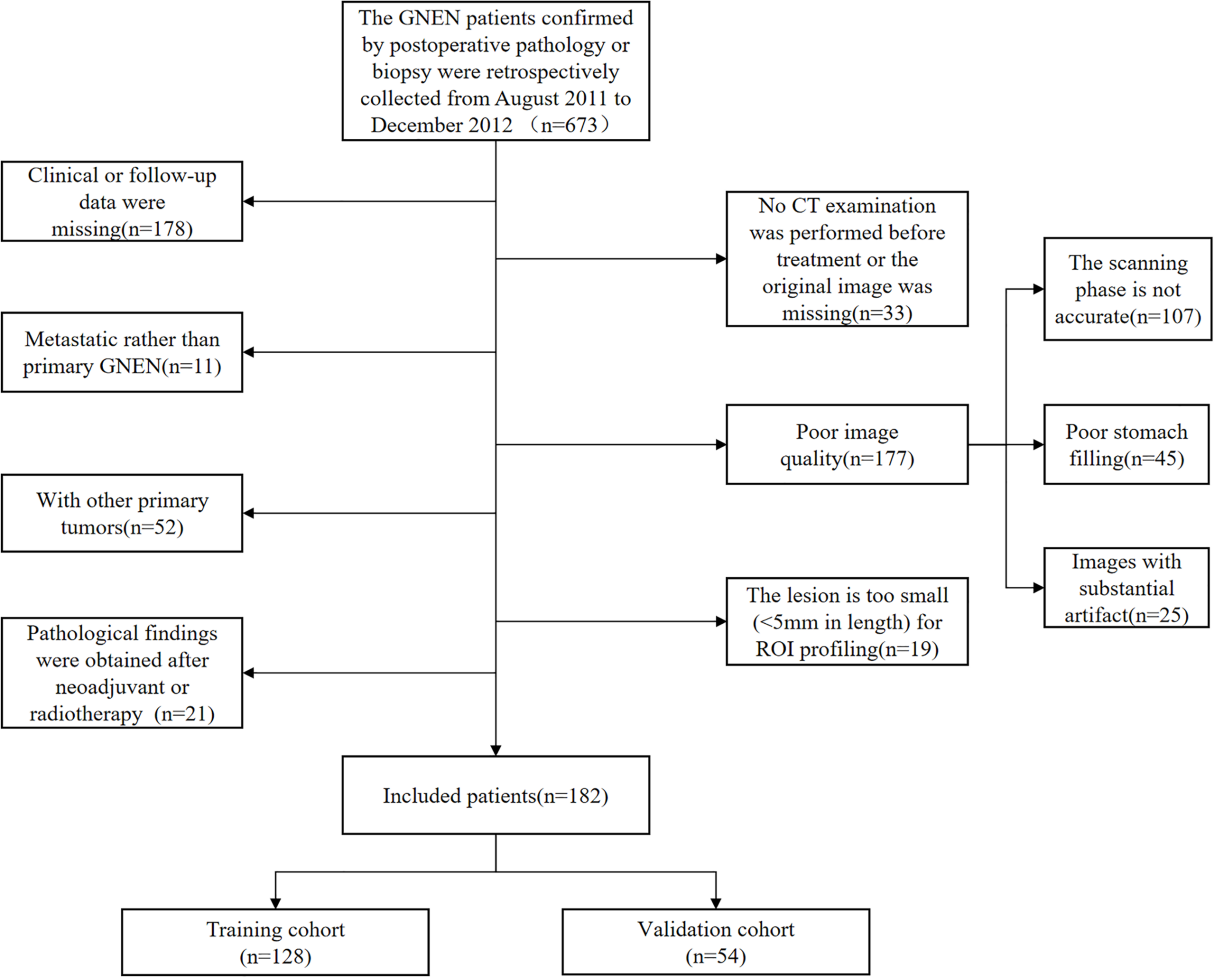
The patient recruitment pathway with inclusion and exclusion details.

The retrospective study was approved by an institutional review board and waived informed consent requirements. A total of 182 GNEN patients who were pathologically confirmed by surgical resection or endoscopic biopsy were enrolled from August 2011 to December 2020. The inclusion criteria were as follows (1): GNEN patients with definite biopsy pathology or postoperative pathological diagnosis; (2) enhanced abdominal CT examination before treatment; (3) complete clinical and pathological data; (4) the follow-up information was complete and reliable. The exclusion criteria were as follows: (1) pathological findings were obtained after neoadjuvant chemotherapy, radiotherapy or other treatments; (2) metastatic GNEN but not primary GNEN; (3) observation and evaluation of the influence of poor CT image quality; (4) the lesion was too small (less than 5mm in length and diameter) to sketch the region of interest (ROI); (5) patients with other primary tumors or severe heart, liver and renal insufficiency. The recruitment process is shown in **Figure 1**.

The patients were randomly divided into training and validation datasets in a 7:3 ratio by computer random number method. Baseline demographic and clinicopathological data were retrospectively extracted from each patient’s electronic medical record. CT image information was obtained from picture archiving and communication system (PACS) and lesion characteristics were recorded.

The follow-up endpoint that this study focused on was OS, which refers to the date of diagnosis to the date of death or the end date of follow-up. Follow-up was conducted every 3 months for the first 2 years, every 6 months for the second to fifth years, and annually thereafter. The end point of follow-up was January 2022. The shortest follow-up time was 12 months and the longest follow-up time was 124 months (mean 29.5 months). Follow-up was conducted by searching inpatient medical records, outpatient return records, or telephone calls to obtain follow-up information.

### CT image acquisition, ROI segmentation, and radiomics feature extraction

The patients were advised to fast for more than 8 hours before examination to ensure gastric emptying. Racemic anisodamine was intramuscularly injected 15 to 20 min before the scan to reduce gastrointestinal peristalsis, and 800 to 1000 mL of warm water was instructed to ensure good gastric filling 5 min before the scan. Perform temporary and effective breathing exercises to reduce the production of respiratory movement artifacts. All patients underwent arterial phase and venous phase enhanced scanning. The details regarding CT image retrieval procedure and the acquisition parameters are described in **Table S1**.

**Figure 2:**
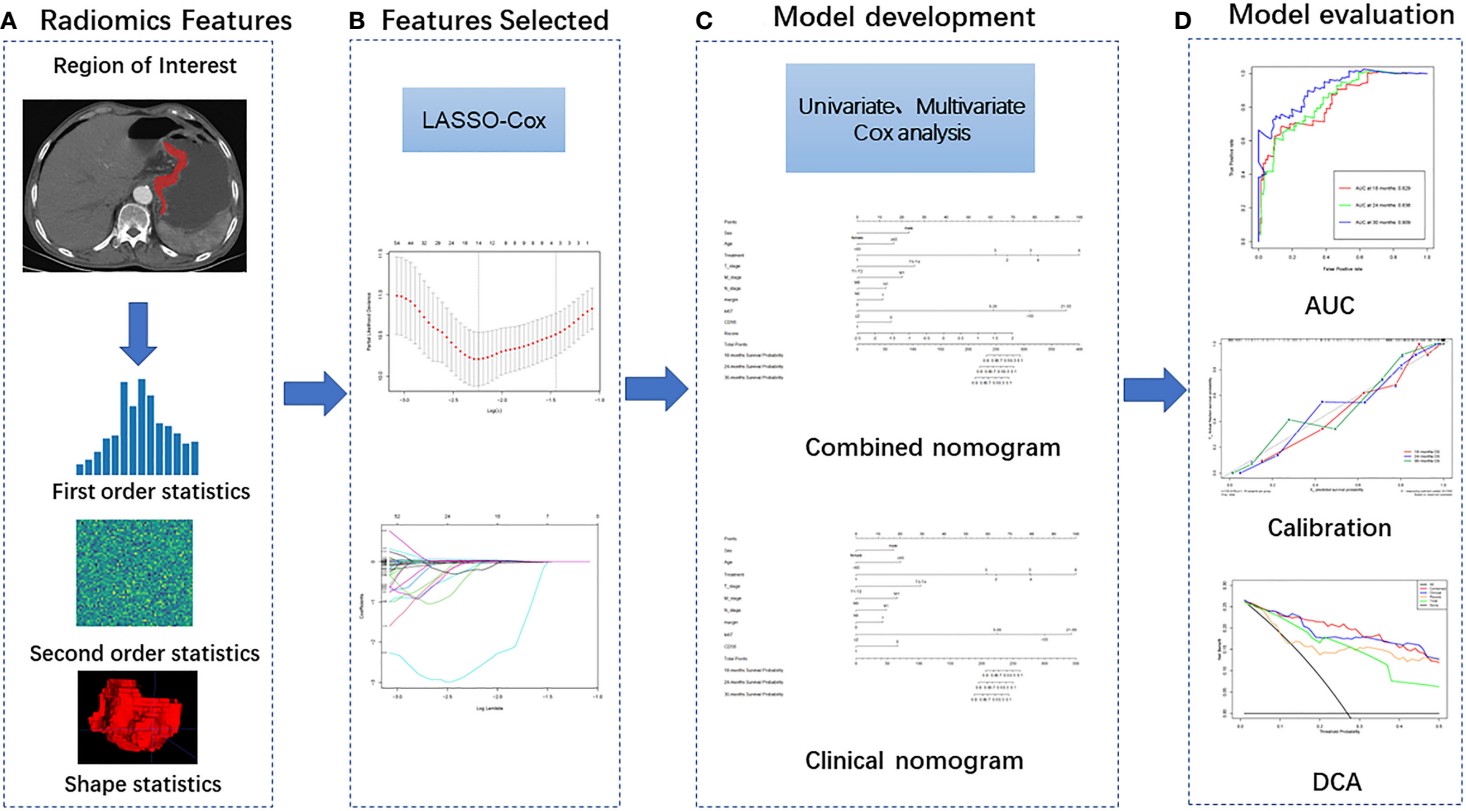
Radiomics workflow including four steps: **(A)** Image segmentation and feature extraction; **(B)** Feature selection; **(C)** Model development and **(D)** Model evaluation.

The radiomics workflow is shown in **Figure 2**. The full sequence target images of arterial phase and venous phase with thickness of 5 mm were downloaded from the PACS system and stored in DICOM format. The three-dimensional ROI was manually outlined layer by layer along the tumor edge in software ITK-SNAP (version 3.8.0, http://www.itksnap.org). Before feature extraction, image resampling and gray discretization were used to standardize the two-phase CT images.

Feature extraction was implemented using PyRadiomics (version 3.0.1) developed by the American Association for Cancer Research. The 1781 radiomics features were extracted from arterial and venous phase respectively, including the following three categories: (1) Shape and size features, which quantitatively describe the geometric characteristics of ROI, such as the three-dimensional diameter, surface area, volume and sphericity of the tumor, which can describe the three-dimensional size and morphology of the tumor; (2) First-order features also known as image intensity features, they are extracted from the histogram information of the tumor region and used to determine the difference between the gray histogram and gray distribution of each ROI; (3) Second-order texture feature, they reflect the spatial arrangement relationship between the gray scale of the image voxel, which are extracted based on the following matrices: Gray Level Co-occurrence Matrix (GLCM), Gray Level Run Length Matrix (GLRLM), Gray Level Dependence Matrix (GLDM), Gray Level Size Zone Matrix (GLSZM), Neighbouring Gray Tone Difference Matrix (NGTDM). In addition, first-order features and second-order texture features were extracted again after filter changes by the following filters: Wavelet, Laplacian of Gaussian (LoG), Square, Square Root, Logarithm, Exponential, Gradient, Local Binary Pattern (2D), Local Binary Pattern (3D).

In order to ensure the stability of the radiomics features, 50 patients were randomly selected one month after the completion of the first ROI segmentation and were segmented by the same radiologist and another radiologist (with 5 and 10 years of image diagnosis experience, respectively). Intra-class and inter-class correlation coefficients were used to evaluate intra-observer and inter-observer repeatability, and radiomics features with both of which >0.75 were retained for subsequent analysis. The first radiologist delineated the remaining ROI.

### Feature selection, development and assessment of the R-signature

The least absolute shrinkage and selection operator (LASSO) penalized Cox proportional hazards regression, which is an accepted algorithm for feature selection in high-dimensional variables was applied to select the optimal prognostic features of the arterial, venous and arteriovenous phases. Then, based on the selected radiomics features of each phase, the R-signature were constructed respectively. Cox proportional risk model was used to calculate the Harrell’s consistency index (C-index) and 95% confidence interval (CI) of the R-signature of each phase, and the R-signature with the best performance of predicting prognosis was selected for subsequent analysis. The C-index, which ranges from 0.5 to 1.0, is commonly used to evaluate the performance of prognostic models in survival analysis. Its higher values revealed that it had greater ability to group patients into different disease progressions. Finally, a multiple-feature-based R-signature, the radiomics score (R-score), was then constructed for predicting survival. A formula was used to calculate the R-score of each individual, which was generated by a linear combination of screening features multiplied by their respective LASSO Cox coefficients. Patients were divided into high risk group and low risk group according to the median R-score of the training group: patients with scores higher than the median were classified as high risk group, and those with scores higher than the median were classified as low risk group. The validation group was grouped with the same cut-off value.

The potential association of the R-signature with OS was first assessed in the training cohort and then validated in the validation cohort by using Kaplan-Meier survival analysis and the difference in survival between the stratified subgroups was determined using the log-rank test. In addition, the prognostic accuracy of the R-score was assessed through the time-dependent receiver operating characteristic (ROC) analysis and the correlated area under the ROC curve (AUC).

### Prognostic model and individualized nomogram analysis

To demonstrate the incremental value of the R-signature relative to the TNM staging system and clinicopathological risk factors for individualized survival assessment of OS, a combined radiomics-clinical model and a purely clinical model were developed in the training cohort. The former combined the radiomics signature and independent clinicopathological risk factors screened based on multivariate Cox analysis, while the latter contained only independent clinicopathological risk factors. Both models were visualized in the form of nomograms. To compare model predicted survival with actual survival, a calibration curve was drawn. In order to quantify the differentiation performance of the models, the C-index values of R-signature, TNM staging system, clinical nomogram and the combined radiomics-clinical nomogram were calculated. Finally, the clinical practicability of each model was determined by comparing the net benefits under different threshold probabilities through decision curve analysis.

### Statistical analysis

Statistical analysis was performed using R software (version 3.1.0) and SPSS software (version 23.0). The continuous variables were first tested for normality. If they fit the normal distribution, they were described in the form of mean standard deviation and compared by the t test of two independent samples. Otherwise, it was described by median (upper and lower quartiles) and compared by Mann-Whitney U test. Classification variables were corrected by Pearson Chi-square test, Chi-square test or Fisher exact test. SPSS was used for univariate and multivariate Cox regression analysis to screen independent risk factors affecting OS. The “glmnet” software package of R software was used for LASSO Cox regression analysis. The “rms” software package was used to generate nomogram and calibration curves, and “dca.R” software package was used for decision curve analysis.

## Results

### Analysis of clinical data

Of the 182 GNEN patients included in the study, 130 (71.4%) were male and 52 (28.6%) were female. The median age (interquartile interval [IQR]) of all patients was 64 years (56-70 years). The treatment methods of all patients were as follows: 23 endoscopic treatments; 46 patients underwent surgical resection; 40 patients received chemotherapy; 10 patients underwent surgical resection after neoadjuvant chemotherapy; 56 patients underwent chemotherapy after surgical resection; another 7 patients were not treated. The median (IQR) survival time for OS in the training cohort was 21.2 (13.46-37.53) months and the mean survival time was 28.94 months. The median survival time (IQR) for OS in the validation cohort was 24.57 (13.75-43.99) months and the mean survival time was 30.91 months. Up to the last follow-up, the number of deaths was 59 (46.0%) in the training cohort and 23 (42.5%) in the validation cohort. The clinicopathological features between the high-risk and low-risk groups in the training and validation cohorts are shown in **Table 1**.

**Table 1:** Clinical features between high - and low-risk groups in training and validation groups.

Characteristics		Training cohort			Testing cohort		
		High risk	Low risk	*P* value	High risk	Low risk	*P* value
**Age**	≥65	37 (57.8)	18 (28.1)	0.001^c^	24 (68.6)	7 (36.8)	0.024^c^
	<65	27 (42.2)	46 (71.9)		11 (31.4)	12 (63.2)	
**Sex**	Male	52 (81.3)	38 (59.4)	0.007^c^	29 (82.9)	11 (57.9)	0.094^d^
	Female	12 (18.7)	26 (40.6)		6 (17.1)	8 (42.1)	
**Symptom**	Abdominal pain and bloating	26 (40.6)	34 (53.2)	0.009^c^	15 (42.9)	34 (53.1)	0.058^e^
	abdominal discomfort	32 (50.0)	15 (23.4)		15 (42.9)	15 (23.5)	
	Hematemesis and black stool	4 (6.3)	7 (10.9)		5 (14.2)	7 (10.9)	
	Physical Findings	2 (3.1)	8 (12.5)		0 (0.0)	8 (12.5)	
**treatment method**	endoscopy	0 (0.0)	17 (26.6)	<0.001^e^	0 (0.0)	6 (31.6)	0.003^e^
	surgery	13 (20.3)	22 (34.4)		6 (17.1)	5 (26.3)	
	chemotherapy	18 (28.1)	6 (9.4)		14 (40.0)	2 (10.5)	
	Neoadjuvant + surgery	7 (10.9)	2 (3.1)		1 (2.9)	0 (0.0)	
	Surgery + chemotherapy	23 (35.9)	16 (25.0)		12 (34.3)	5 (26.3)	
	untreated	3 (4.7)	1 (1.6)		2 (5.7)	1 (5.3)	
**Location**	Cardia/fundus	42 (65.6)	23 (35.9)	<0.001^c^	21 (60.0)	10 (52.6)	0.019^e^
	Body	9 (14.1)	29 (45.3)		9 (25.7)	5 (26.3)	
	Antrum	2 (3.1)	10 (15.6)		0 (0.0)	4 (21.1)	
	≥2/3 stomach	11 (17.2)	2 (3.1)		5 (14.3)	0 (0.0)	
**Margin**	unclear	44 (68.7)	20 (31.3)	<0.001^c^	26 (74.3)	8 (42.1)	0.019^c^
	clear	20 (31.3)	44 (68.7)		9 (25.7)	11 (57.9)	
**Cystic/necrosis**	Yes	29 (45.3)	9 (14.1)	<0.001^c^	21 (60.0)	7 (36.8)	0.104^c^
	No	35 (54.7)	55 (85.9)		14 (40.0)	12 (63.2)	
**ulceration**	Yes	47 (73.4)	24 (37.5)	<0.001^c^	25 (71.4)	7 (36.8)	0.014^c^
	No	17 (26.6)	40 (62.5)		10 (28.6)	12 (63.2)	
**T stage**	T3~4	59 (92.2)	24 (37.5)	<0.001^c^	33 (94.3)	8 (42.1)	0.000^d^
	T1~2	5 (7.8)	40 (62.5)		2 (5.7)	11 (57.9)	
**N stage**	N1	48 (75.0)	13 (20.3)	<0.001^c^	24 (68.6)	5 (26.3)	0.003^c^
	N0	16 (25.0)	51 (79.7)		11 (31.4)	14 (73.7)	
**M stage**	M1	16 (25.0)	4 (6.3)	0.003^c^	12 (34.3)	3 (15.8)	0.147^c^
	M0	48 (75.0)	60 (93.8)		23 (65.7)	16 (84.2)	
**CT_AP_**		66.49 (59.00~ 79.74)	67.00 (53.27~ 87.00)	0.977^b^	63.33 (59.88~ 71.40)	76.00 (58.00~ 98.58)	0.163^b^
**CT_VP_**		77.10 (67.18~ 87.86)	71.39 (61.21~ 97.59)	0.617^b^	78.19 ± 16.66	86.16 ± 25.61	0.232^a^
**Longest diameter**		61.67 (48.64~ 78.80)	29.18 (11.94~ 40.39)	<0.001^b^	54.94 (44.27~ 61.98)	31.22 (22.30~ 47.74)	<0.001^b^
**Thickest diameter**		20.21 (15.29~ 28.53)	12.80 (9.18~ 16.07)	<0.001^b^	18.59 (14.31~ 24.38)	13.78 (10.82~ 18.40)	<0.001^b^
**Ki67**	≤2	0 (0.0)	19 (29.7)	<0.001e	0 (0.0)	3 (15.8)	<0.001e
	3~20	0 (0.0)	13 (20.3)		2 (5.7)	8 (42.1)	
	21~55	4 (6.3)	4 (6.3)		5 (14.3)	1 (5.3)	
	>55	60 (93.8)	28 (43.8)		28 (80.0)	7 (36.8)	
**Grade**	NETG1	0 (0.0)	19 (29.7)	<0.001e	0 (0.0)	4 (21.1)	<0.001e
	NETG2	0 (0.0)	12 (18.8)		1 (2.9)	7 (36.8)	
	NETG3	2 (3.1)	3 (4.7)		3 (8.6)	0 (0.0)	
	MiNEN	13 (20.3)	13 (20.3)		7 (20.0)	3 (15.8)	
	NEC	49 (76.6)	17 (26.6)		24 (68.6)	5 (26.3)	
**Syn**	Negative	1 (1.6)	2 (3.1)	1.000d	1 (2.9)	1 (5.3)	1.000d
	Positive	63 (98.4)	62 (96.9)		34 (97.1)	18 (94.7)	
**CD56**	Negative	12 (18.8)	8 (12.5)	0.330c	5 (14.3)	1 (5.3)	0.579d

a, t test; b, Mann-Whitney U test; c, Pearson Chi-square test; d, calibration chi-square test; e, Fisher exact test.

### Construction of the regression model based on R-signature

Repeatable and stable radiomics features (inter class and intra class correlation coefficient>0.75) were retained. Through LASSO Cox dimension reduction screening for features of each phase(**Figure S1A, B**), 14 radiomics features were finally screened in arterial phase (**Table S2**), 10 radiomics features were screened in venous phase (**Table S3**), and 14 radiomics features were screened in the combined arteriovenous phase (**Table S4**). Based on the screened features of each phase, corresponding R-signature were constructed respectively, and the performance of OS prediction of each phase was compared by calculating the C-index values. The results showed that the R-signature of combined arteriovenous phase was better than that of arterial phase and venous phase(**Table 2**). Therefore, it was applied in the next analysis. The R-score of the optimal phase was then calculated according to the formula (**Supplemental Material**). The cut-off value of the R-score was −0.4939. Consequently, patients were stratified into high-risk group (R-score ≥ −0.4939) and low-risk group (R-score < −0.4939).

**Table 2:** C-index of the R-signature in each phase.

Model	OS	
	C-index value (95%CI)	*P* value
Training cohort		
Arterial phase R-signature	0.784 (0.761~0.868)	<0.001
Venous phase R-signature	0.756 (0.735~0.857)	<0.001
Arteriovenous phase combined R-signature	0.803 (0.741~0.864)	<0.001
Validation cohort		
Arterial phase R-signature	0.747 (0.651~0.843)	<0.001
Venous phase R-signature	0.723 (0.681~0.845)	<0.001
Arteriovenous phase combined R-signature	0.751 (0.661~0.841)	<0.001

### Validation of the predictive utility of the R-signature

**Figure 3:**
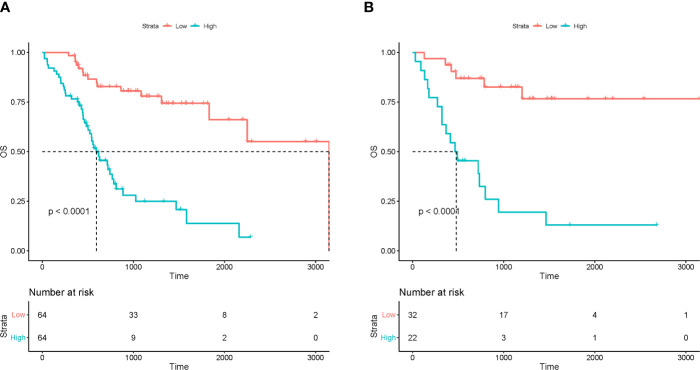
The Kaplan-Meier curves for patients in low-risk and high-risk groups in the training **(A)** and test **(B)** cohorts.

Kaplan-Meier survival analysis showed that R-signature based on multiple radiomics features were significantly correlated with OS in the training cohort [hazard ratio (HR) =5.015; 95%CI 3.602~5.982, P<0.001] (**Figure 3A**). The OS of the low-risk group was 95.3% at 12 months, 86.5% at 18 months, 82.8% at 24 months, and 80.6% at 30 months. The OS of the high-risk group was 76.5% at 12 months, 57.2% at 18 months, 41.0% at 24 months, and 28.0% at 30 months. The low-risk group has a better OS compared to a high-risk group (P < 0.001, log-rank test). Subsequently, similar results were observed in the validation cohort, where R-signature was also significantly correlated with OS (HR=6.829; 95% CI 2.661 to 17.527, P< 0.001) (**Figure 3B**). In the low-risk group, the OS was 93.8% at 12 months, 86.9% at 18 months, 86.9% at 24 months and 82.6% at 30 months. In the high-risk group, the OS was 63.6% at 12 months, 45.5% at 18 months, 39.0% at 24 months and 26.0% at 30 months. And the low-risk group was likewise associated with better OS (P < 0.001, log-rank test).

**Figure 4:**
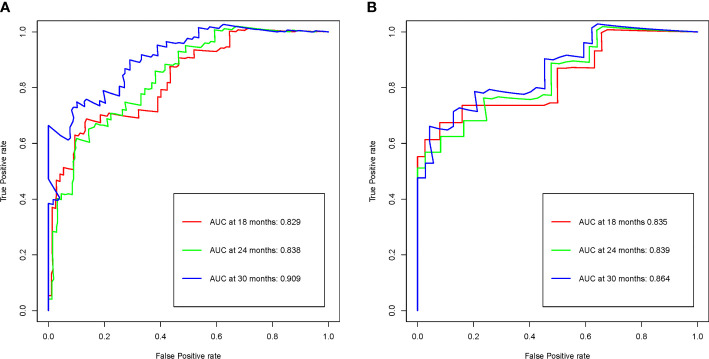
R-signature evaluated by time-dependent ROC curves in the training **(A)** and validation **(B)** cohorts.

Time-dependent ROC analysis was used to evaluate the prognostic accuracy of the R-signature in both the training cohort and the validation cohort. The results showed that in the training cohort, the AUC values for 18-month OS, 24-month OS and 30-month were 0.829, 0.838 and 0.909 respectively (**Figure 4A**). In the validation cohort, the AUC values for 18-month OS, 24-month OS and 30-month OS were 0.835, 0.839 and 0.864, respectively (**Figure 4B**). The above data proved the discrimination accuracy of OS was reliable and robust when using R-score.

### Performance of R-signature in individualized nomogram with OS prediction

**Figure 5:**
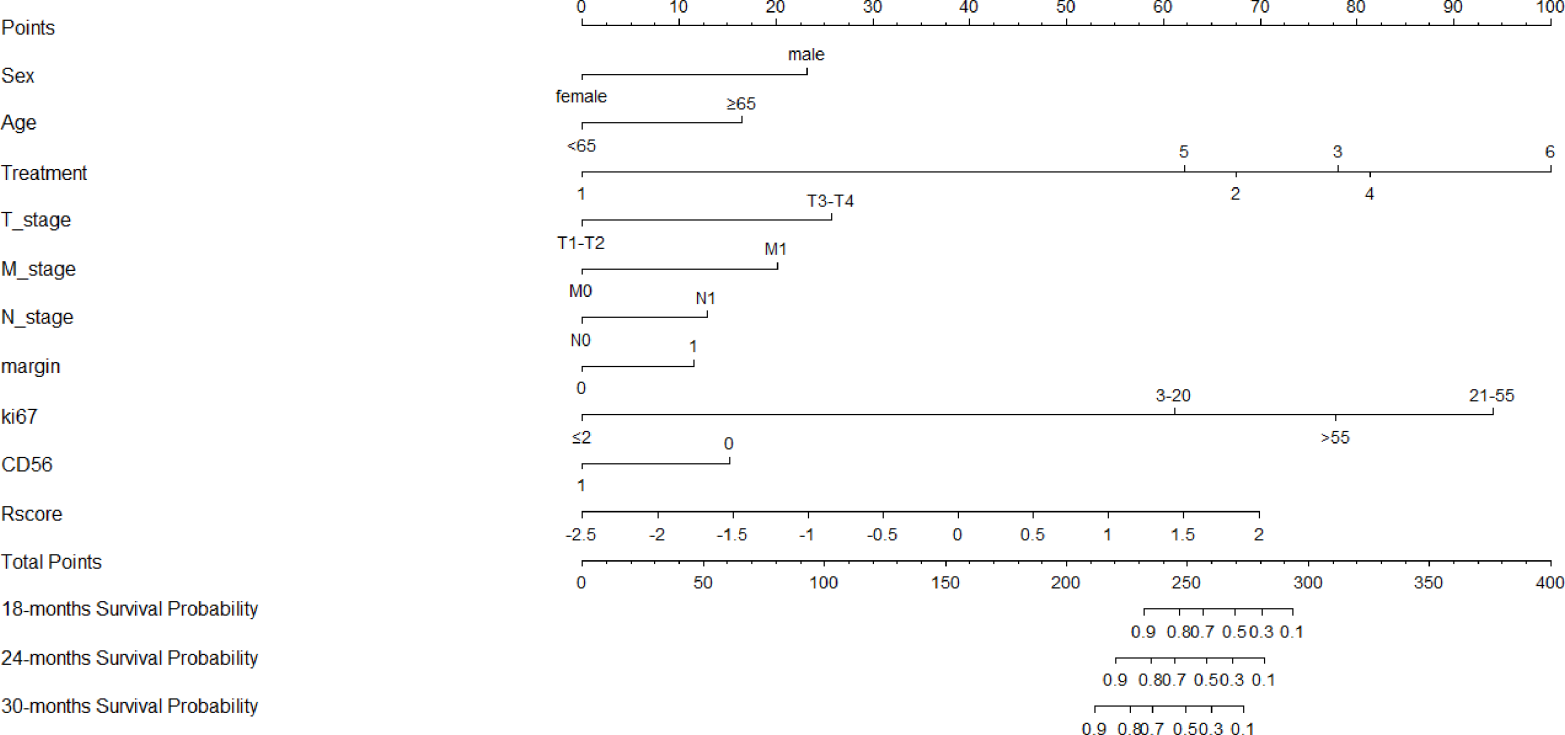
Nomogram of the combined radiomics-clinical model for predicting OS, which showed the weight of each variable when Rscore and clinicopathological risk factors were included.

**Figure 6:**
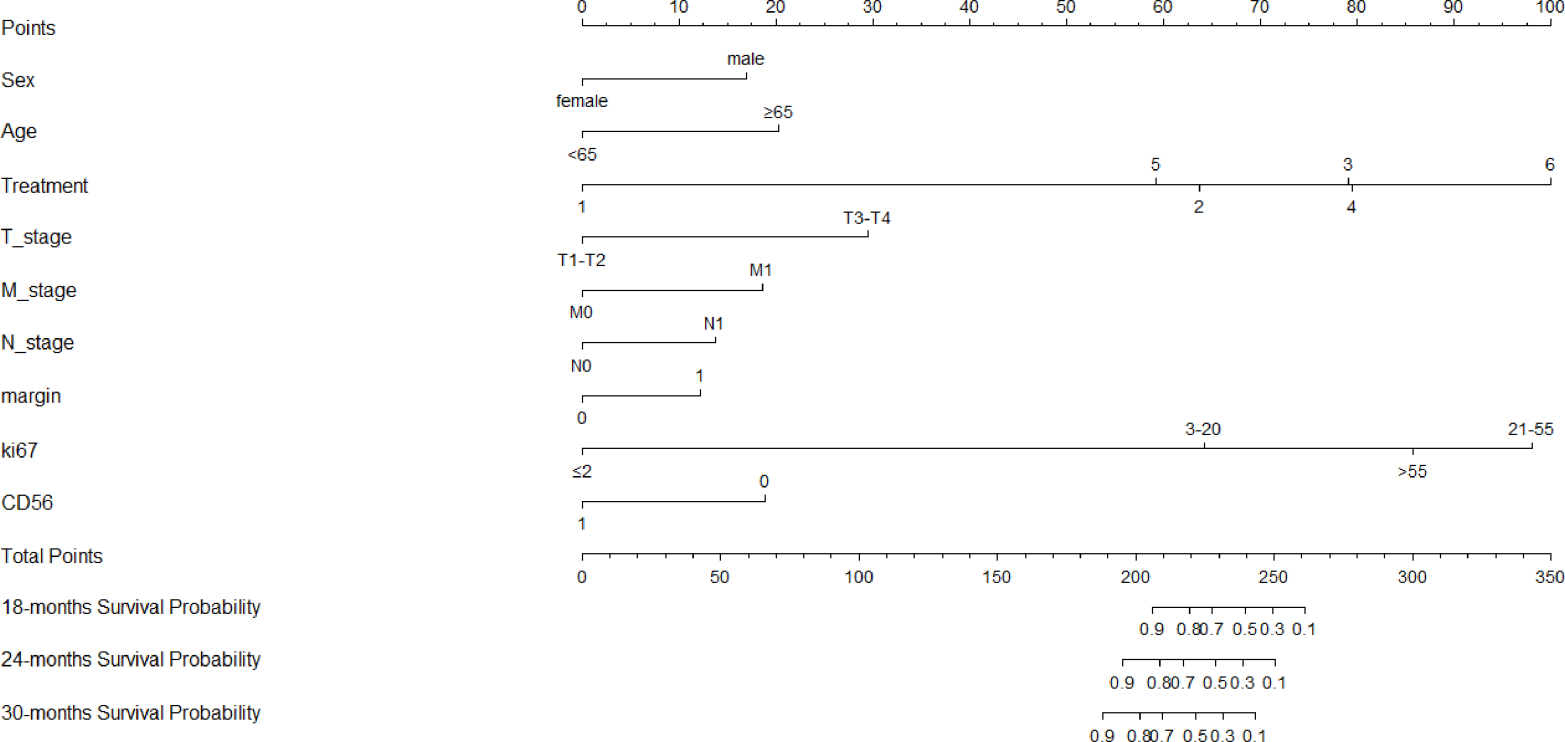
Nomogram of the clinical model for predicting OS, which showed the weight of included clinicopathological risk factors.

**Figure 7:**
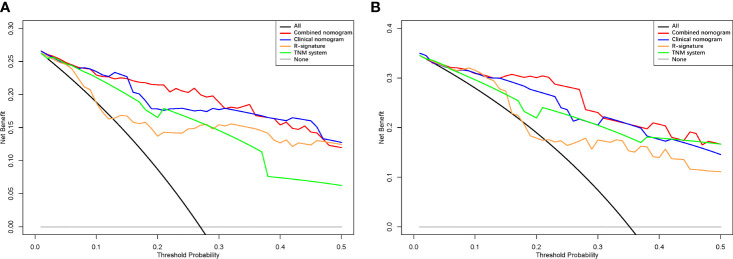
Decision curve analysis of each model in the training **(A)** and validation **(B)** cohorts.

Clinicopathological factors were selected by univariate Cox regression analysis (**Table 3**), and the factors with P<0.05 were again included in the multivariate Cox regression (**Table 4**). By stepwise backward regression, we found that R-score was a strong independent prognostic factor for OS, and gender, age, treatment methods, T stage, N stage, M stage, tumor boundary, Ki67, CD56 were also independent risk factors affecting prognosis (P<0.05). Combined with R-score and clinicopathological factors, the combined radiomics-clinical model was constructed, and the individualized prediction model of OS was further visualized as nomogram (**Figure 5**). In addition, clinical model were independently constructed using clinicopathological risk factors, which was also shown in the form of nomogram (**Figure 6**). **Figure S2A, B** depicted the calibration curves of the combined radiomics-clinical model for predicting OS at 18 months, 24 months and 30 months. In the training cohort and the validation cohort, the estimated values predicted by the model were in good agreement with the actual observed values. **Figures S3A, B** depicted the calibration curves of 18, 24 and 30 months OS predicted by the clinical model. In the training and validation cohorts, the estimated values predicted by the model were also very consistent with the actual observed values. By calculating the C-index value of each model, we found that compared with TNM staging system or clinical nomogram and R-signature, the combined radiomics-clinical nomogram showed better discrimination ability in the training and validation cohorts, and the prediction ability of the model was improved (C-index=0.882; 95% CI 0.848~0.916) (**Table 5**). Decision curve analysis showed that the overall net benefit of the combined radiomics-clinical nomogram was higher than that of the clinical nomogram, R-signature and the TNM staging system in most reasonable threshold probability ranges (**Figure 7**).

**Table 3:** Univariate Cox regression analysis.

Features		Univariate Cox	
		HR (95%CI)	*P* value
**R-score**		5.015 (3.602~6.982)	0.000
**Age**	≥65	2.505 (1.539~3.938)	0.000
	<65	1.000	
**Sex**	Male	3.031 (1.642~5.597)	0.000
	Female	1.000	
**Symptom**	Abdominal pain and bloating	2.146 (0.659~6.985)	0.205
	Abdominal discomfort	2.943 (0.905~9.568)	0.073
	Hematemesis and black stool	3.153 (0.852~11.659)	0.085
	Physical Findings	1.000	0.190
**Treatment method**	Endoscopy	0.013 (0.002~0.117)	0.000
	Surgery	0.153 (0.055~0.428)	0.000
	Chemotherapy	0.760 (0.295~1.959)	0.570
	Neoadjuvant + surgery	0.279 (0.075~1.042)	0.058
	Surgery + chemotherapy	0.159 (0.058~0.433)	0.000
	Untreated	1.000	
**Location**	Cardia/fundus	0.990 (0.486~2.018)	0.978
	Body	0.466 (0.205~1.058)	0.068
	Antrum	0.531 (0.177~1.589)	0.257
	4≥2/3 stomach	1.000	0.042
**Margin**	Unclear	1.734 (1.110~2.708)	0.016
	Clear	1.000	
**Cystic/necrosis**	Yes	2.351 (1.522~3.630)	0.000
	No	1.000	
**Ulceration**	Yes	2.435 (1.502~3.947)	0.000
	No	1.000	
**T stage**	T3~4	9.522 (4.135~21.929)	0.000
	T1~2	1.000	
**N stage**	N1	4.711 (2.881~7.701)	0.000
	N0	1.000	
**M stage**	M1	5.348 (3.335~8.575)	0.000
	M0	1.000	
**CT_AP_**		0.993 (0.983~1.003)	0.147
**CT_VP_**		0.998 (0.989~1.007)	0.702
**Longest diameter**		1.015 (1.009~1.020)	0.000
**Thickest diameter**		1.041 (1.025~1.057)	0.000
**Ki67**	≤2	0.048 (0.007~0.347)	0.003
	3~20	0.079 (0.019~0.322)	0.000
	21~55	1.059 (0.486~2.308)	0.886
	>55	1.000	
**Grade**	NET G1	0.035 (0.005~0.253)	0.001
	NET G2	0.042 (0.006~0.304)	0.002
	NETG3	0.965 (0.387~2.408)	0.939
	MiNEN	0.462 (0.248~0.859)	0.015
	NEC	1.000	
**Syn**	Negative	1.101 (0.270~4.484)	0.894
	Positive	1.000	
**CD56**	Negative	1.717 (1.001~2.946)	0.050
	Positive	1.000	

**Table 4:** Multivariate Cox regression analysis.

Variable	Coefficient of covariate	Standard error	Wald	Degree of freedom	Significance	HR (95%CI)
**Age**	0.573	0.288	3.951	1	0.047	1.774 (1.008~3.123)
**Sex**	0.834	0.372	5.021	1	0.025	2.304 (1.110~4.779)
**treatment method**			12.248	5	0.032	
endoscopy	-0.547	1.845	0.088	1	0.767	0.579 (0.016~21.512)
surgery	-1.432	0.611	5.490	1	0.019	0.239 (0.072~0.791)
chemotherapy	-1.037	0.551	3.537	1	0.060	0.355 (0.120~1.045)
Neoadjuvant + surgery	-1.623	0.725	5.014	1	0.025	0.197 (0.048~0.817)
Surgery + chemotherapy	-1.939	0.612	10.051	1	0.002	0.144 (0.043~0.477)
**Margin**	-0.503	0.258	3.799	1	0.049	0.605 (0.365~1.003)
**T stage**	1.355	0.508	7.125	1	0.008	3.876 (1.433~10.479)
**N stage**	0.524	0.269	3.802	1	0.048	1.688 (0.997~2.859)
**M stage**	0.997	0.314	10.070	1	0.002	2.711 (1.464~5.018)
**ki67**			5.552	3	0.136	
≤2	-0.644	1.785	0.130	1	0.718	0.525 (0.016~17.367)
3~20	-1.618	0.775	4.357	1	0.037	0.198 (0.043~0.906)
21~55	0.450	0.449	1.007	1	0.316	1.569 (0.651~3.781)
**CD56**	-0.907	0.309	8.601	1	0.003	1.404 (1.220~1.740)
**R-score**	0.901	0.239	14.258	1	0.000	2.462 (1.543~3.931)

**Table 5:** C-index values of different models.

Model	OS	
	C-index value (95%CI)	*P* value
Training cohort		
R-signature	0.803 (0.741~0.864)	<0.001
TNM staging system	0.870 (0.815~0.925)	<0.001
Clinical nomogram	0.861 (0.819~0.903)	<0.001
Combined radiomics-clinical nomogram	0.882 (0.848~0.916)	<0.001
Validation cohort		
R-signature	0.751 (0.661~0.841)	<0.001
TNM staging system	0.801 (0.689~0.913)	<0.001
Clinical nomogram	0.796 (0.722~0.871)	<0.001
Combined radiomics-clinical nomogram	0.827 (0.765~0.888)	<0.001

## Discussion

GNEN is a group of rare, heterogeneous tumors with diverse clinical behavior, histomorphology, and genomic features that have steadily increased in incidence and prevalence over the past few decades (2). Dasari A et al. evaluated the prognosis of 64971 NEN patients from the SEER database, and the results showed that G1 grade undifferentiated tumors had the highest median OS (16.2 years), G2 grade differentiated tumors had poor OS (8.3 years), and G3 grade poorly differentiated tumors had the worst OS (10 months) (2). Therefore, it is critical to accurately predict the prognosis of GNEN due to the heterogeneous prognosis of patients. However, in actual clinical work, there are two different TNM staging systems for GNET and GNEC, which have become a major problem plaguing clinicians, and there are still a large number of defects in personalized evaluation of GNEN prognosis.

In recent years, some researchers have established nomogram models to predict the prognosis of gastrointestinal and pancreatic NEN (GEP NEN) and GNEC patients, and have achieved good predictive effect (22, 23). They were based on TNM staging system and some clinical factors. However, TNM staging based on tumor anatomical characteristics could not reflect the innate biological heterogeneity of tumors, and was not sufficient to provide complete and accurate prognostic information. Fortunately, the radiomics approach provides a robust and non-invasive method for characterizing intratumor heterogeneity by non-invasive extraction of whole-tumor characterization (24). Previous studies have reported that proteomic and phenotypic information can be inferred from radiological images of tumors, which were often associated with tumor recurrence and metastasis and thus may be key prognostic biomarkers (25, 26). Our team’s previous research found that radiomics nomograms had important clinical significance for preoperative detection of gastric malignancies, and radiomics analysis had shown good performance in distinguishing GNEC from gastric adenocarcinoma (27). However, current radiomics studies on the prognosis of GNEN patients are still lacking. Therefore, this study aimed to develop and validate a novel CT-based prognostic R-signature for use in conjunction with the TNM staging system to improve the prediction of OS.

In this study, we found that the combination of R-signature and clinicopathology data showed a higher predictive value than the clinical nomogram or R-signature alone, thus suggesting that it may be used to predict OS in patients with GNEN. The study took into account valuable clinical factors. According to our nomogram, male patients over 65 years old may had a higher disease specific death prediction than young female patients. This was basically consistent with the clinical nomogram established by Fang et al. suggesting that patients over 50 years old had a higher probability of disease-related mortality than younger patients (22). For female patients, high hormone level fluctuations lead to a low degree of physical discomfort, so timely intervention may be associated with a better prognosis.

We also found that whether the margin was clear or not had an effect on prognosis (p = 0.016), this was different from Yan’s results [15]. In their study, no significant difference was found in OS between the group with clear margin and the group with unclear margin. This may be due to the differences in sample size and the proportion of samples with unclear margin. In our study, 182 cases were included, more than twice as many of them, and 53.8% (98/182) of our cases had unclear margin. In contrast, only 14.5% (11/76) cases had unclear margin in their study. This disparity in sample size, as well as differences in the proportion of subgroups, may lead to the difference between our results. What’s more, the symptom was proved not a significant factor for OS. This phenomenon has not been found in other studies.

Due to GEP-NEN’s rarity, complexity and poor understanding of the disease, there is still a lack of a unified treatment approach, and GEP-NEN treatment should be highly personalized. Endoscopic mucosal dissection is recommended for GEP-NEN limited to mucosa or submucosa with a length of no more than 1 cm G1 grade and no lymph node metastasis or distant metastasis, with a lesion resection rate of more than 85% and a good prognosis. Furthermore, AJCC guidelines recommend palliative chemotherapy for patients with poor histological differentiation because their life expectancy is very low. Patients with good histological differentiation have a better prognosis, so surgical resection of the lesion may be considered (28). Surgical operations are divided into radical resection and palliative resection. Many scholars recommend radical resection for patients with GNEC to prolong survival time, because the 5-year and 3-year OS of the former were higher than those of the latter (6, 29). ENETS guidelines recommend adjuvant chemotherapy for patients with advanced GNEC, and some studies have also shown that postoperative adjuvant chemotherapy can improve the survival of patients with NEC (30–32). In line with this, our nomogram intuitively reflected that postoperative adjuvant chemotherapy had a lower score than surgery alone, and therefore a higher predicted survival rate. Moreover, we found that active treatment was a protective factor for the prognosis of patients through multivariate Cox regression analysis. As for the comparison of prognostic differences among various treatments, further studies should be conducted after further unification of baseline data and enlargement of sample size.

Ki⁃67 index is an important indicator of tumor proliferation activity (33). Sorby et al. reported that patients with Ki67 index>55% had better response to platinum based chemotherapy, but shorter survival period (10). Boo et al. found that high Ki67 proliferation index (>60%) was related to tumor recurrence and tissue differentiation, which can be used as a prognostic indicator of GNEC (34). Xie et al. showed that when the Ki67 proliferation index increased, the survival rate of patients decreased significantly (6). However, the Ki67 index alone does not seem to be the most important parameter in determining the potential for metastasis, as gastric tumor property was also closely related to tumor size and the depth of gastric wall tumor invasion (35). This study analyzed the impact of Ki67 proliferation index on the prognosis of patients. According to the recommendations of previous literature, patients were divided into four categories: Ki67<2%, 3% - 20%, 20% - 55%, and>55%, corresponding to NET G1, NET G2, NET G3 and some NEC, and highly malignant NEC patients with poor differentiation (3). After univariate and multivariate Cox regression analysis, it was confirmed that Ki67 index was an independent risk factor affecting the prognosis.

Synaptophysin (Syn), Chrogranin A (CgA), and CD56 are three neuroendocrine markers widely used in the diagnosis of NEN (36). In patients included in this study, Syn and CgA were missing more data, and only CD56 with complete data was discussed. In this study, the positive expression of CD56 accounted for 85.7%. After multivariate regression analysis, it was found that negative CD56 was a risk factor for prognosis.

In addition, our study showed that patients with lymph node metastasis and distant metastasis were more likely to die than patients without metastasis. The nomogram also illustrated that the prognosis of patients with T3-4 was worse than that of patients with T1-2. The above findings have also been reported in previous literature. A Chinese study involving 419 patients with rectal NEN showed that lymph node metastasis was an important risk factor for the prognosis of rectal NEN (37). Two other studies have also shown that lymph node metastasis and distant metastasis are independent covariates associated with OS in GNEN and GEP-NEN, respectively (22, 38). Yan et al. proposed that serous infiltration and lymph node metastasis were independent risk factors for disease free survival (DFS) and OS in GNEN patients, respectively (15).

In this study, although tumor boundary was an independent risk factor affecting prognosis, it had a weak influence on prognosis, with a covariate coefficient of only -0.503. Accordingly, it also accounted for a small assigned score in nomogram. Similarly, it has been reported that boundary blurring was associated with high recurrence rate and poor survival rate in pancreatic neuroendocrine tumor (39).

The prognosis of patients with GNEN depends on a complex multi-factor interaction, and a single but powerful independent risk factor is not sufficient to accurately predict the prognosis. In addition, the single factor only applies at the population level, not the individual level. While synthetic nomogram provides an accurate and objective prognosis and facilitates personalized treatment based on the patient’s specific situation. Previous studies have shown that nomogram shows better performance in predicting survival in NEN patients compared to the traditional TNM staging system (23, 28). In this study, the predictive performance of the arterial phase radiomics model was superior to that of the venous phase radiomics model, while the performance of the arteriovenous phase combined radiomics model was superior to any single phase imaging model. Similar findings were also found in previous studies, which may be due to the fact that the combined phase provided more abundant radiomics features than the independent phase (40, 41). In addition, GNEN is characterized by significant enhancement in the arterial phase, which reflects the characteristics of tumor blood supply and functional capillaries. Meanwhile, angiogenesis is closely related to the occurrence, development and prognosis of tumors. We further combined the arteriovenous phase combined R- signature with independent clinicopathological risk factors to develop a combined radiomics-clinical nomogram to predict OS probability at 18, 24, and 30 months for individual patients. Decision curve analysis showed that, in most reasonable threshold probability ranges, the combined radiomics-clinical nomogram was superior to the clinical nomogram and TNM staging system, indicating that the R-signature provided supplementary prognostic information, improved the prognostic performance of the TNM staging system, and provided incremental value for individualized prognostic evaluation.

There were several limitations to this study. First of all, this study was a retrospective study, and the disease was relatively rare, so the sample size included was small. Secondly, the clinical efficacy of our nomogram needs to be validated through prospective, multi-center collection of external data to improve the generalization ability. Finally, this study only focused on the prognostic outcome of OS, and did not discuss the clinical value of radiomics signature for progression-free survival (PFS), which is the direction of further research.

## Conclusions

In summary, our findings demonstrated that R-signature could be used to stratify GNEN patients into high and low risk groups. Moreover, the newly developed combined radiomics-clinical nomogram is a powerful predictor of OS for GNEN patients, which demonstrated incremental value of the R-signature to the traditional staging system for individualized survival estimation.

## Data availability statement

The original contributions presented in the study are included in the article/**Supplementary Material**. Further inquiries can be directed to the corresponding author.

## Author contributions

Z-HY: design the research. Y-JH: performed the research. Y-JH, RW and JL: collected the data. MC and H-PZ: analyzed the data. Z-HY and Y-JH: analyzed the data and wrote the paper. MC and J-BG: reviewed the paper. All authors contributed to the article and approved the submitted version.
