# Supplementary material for: Prognostic value of computed tomography radiomics features in patients with gastric neuroendocrine neoplasm
Source: Front Oncol. 2023 Jun 20;13:1143291. doi: 10.3389/fonc.2023.1143291 (PMC10319063; doi:10.3389/fonc.2023.1143291)
Supplement: Supplementary file 1 [file DataSheet_1.docx]

**Supplementary Figure 1：Feature selection for the prediction of OS using the LASSO Cox regression model in arteriovenous combined phase (A) Tuning parameter selection in the LASSO model involved the use of tenfold cross-validation with minimum criteria. (B) We examined the coefficients to identify potential predictors.**

**
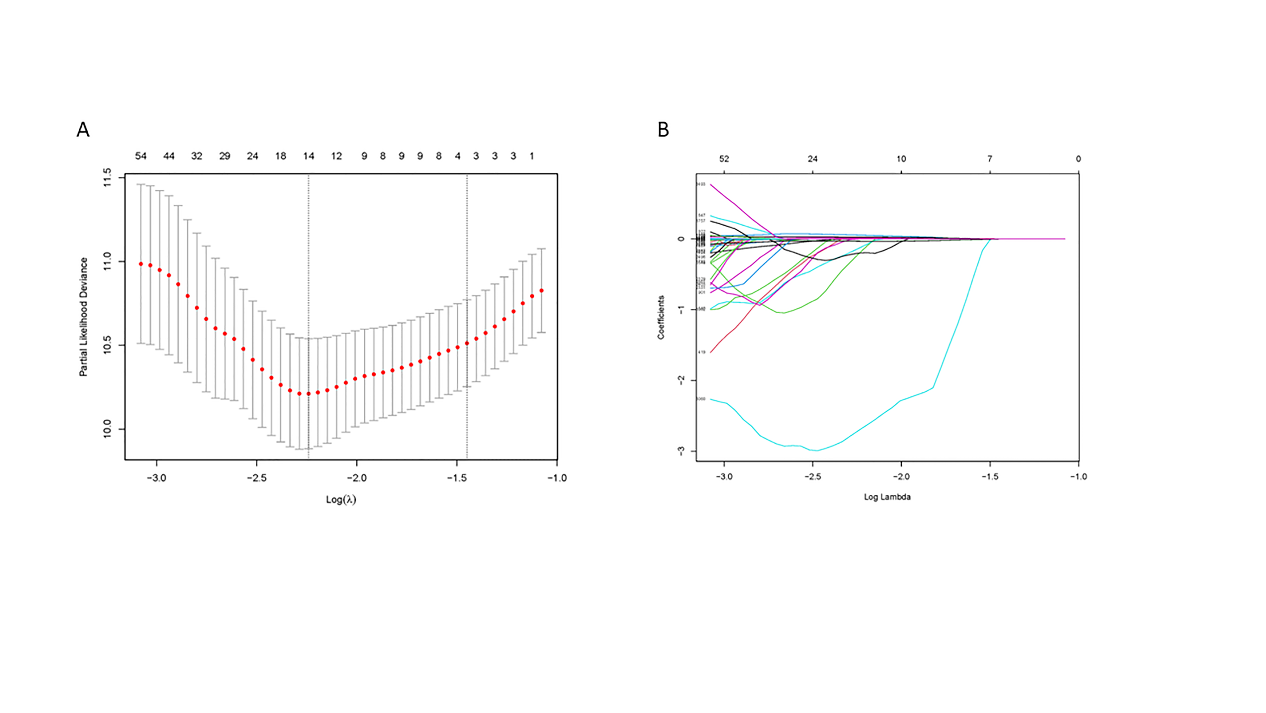
**

**Supplementary Figure 2: Calibration curve of the training(A) and validation (B) cohorts in the combined radiomics-clinical model which based on the correspondence between predicted OS and observed OS at 18, 24, and 30 months.**

**
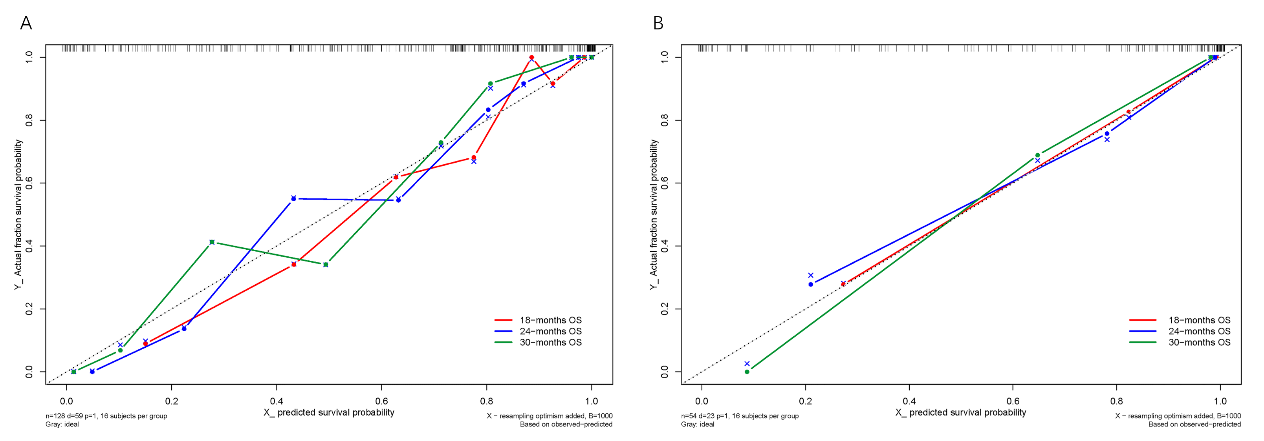
**

**Supplementary Figure 3: Calibration curve of the training(A) and validation (B) cohorts in clinical model which based on the correspondence between predicted OS and observed OS at 18, 24, and 30 months.**

**
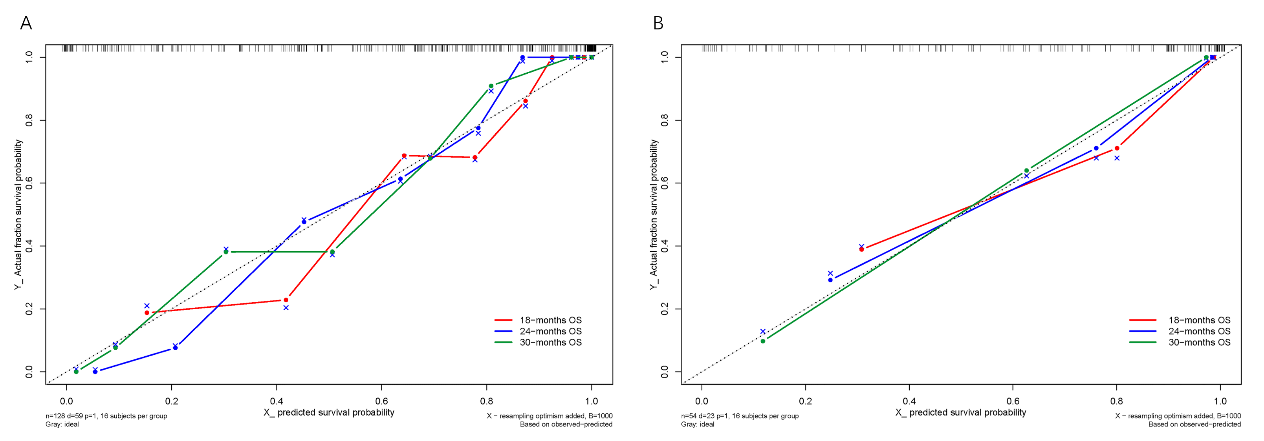
**

**Supplementary Table 1**

**CT scanning equipment and scanning scheme**

|  | **Parameters** | **Scheme** |
| --- | --- | --- |
| **Equipment** | Equipment Name | GE Discovery 750 HD CT |
|  |  | Philips Brilliance iCT |
|  |  | GE BrightSpeed CT |
|  |  | Simens Somatom Perspective CT |
| **Scanned protocol** | Width of collimator | 32×0.6 mm or 64×0.625 mm |
|  | Rotation time | 0.5~0.8 s/r |
|  | Tube Voltage | 120 kVp |
|  | Tube current | 290~650 mA |
|  | Pitch | 1.375:1/0.992:1 |
|  | Layer thickness/ spacing | 5.0 mm/5.0 mm |
|  | Matrix | 512×512 |
|  | Noise figure | 10 HU |
| **Scanned area** | Location standard | Top of diaphragm to lower pole of both kidneys |
| **Enhancement**  **Condition** | Contrast agent | iohexol：300 mgI/mL or ioversol：320 mg/ml |
|  | Flow rate | 2.5~3.5 ml/s |
|  | Dose | 1.5 ml/kg |
|  | Acquisition time | arterial phase：30s；venous phase：60~70s |
| **Postprocess** | reconstruction thickness | 0.625 mm、1.25 mm |

**Supplementary Table 2**

**Radiomics characteristics in arterial phase**

| Features | Coefficient | Exponential coefficient |
| --- | --- | --- |
| A_wavelet.LLH_glcm_Imc2 | -1.0601 | 0.3464 |
| A_wavelet.HLL_glcm_Imc2 | -0.5707 | 0.5651 |
| A_log.sigma.2.0.mm.3D_glrlm_ShortRunLowGrayLevelEmphasis | -0.5246 | 0.5918 |
| A_original_shape_Sphericity | -0.5093 | 0.6009 |
| A_logarithm_glcm_MCC | -0.1485 | 0.8620 |
| A_wavelet.HLL_firstorder_Skewness | -0.0366 | 0.9641 |
| A_square_glszm_GrayLevelNonUniformity | -0.0239 | 0.9764 |
| A_squareroot_firstorder_Maximum | 0.0008 | 1.0008 |
| A_logarithm_firstorder_Mean | 0.0011 | 1.0011 |
| A_original_shape_Maximum2DDiameterColumn | 0.0030 | 1.0030 |
| A_original_shape_Maximum2DdiameterSlice | 0.0050 | 1.0050 |
| A_log.sigma.4.0.mm.3D_glszm_GrayLevelNonUniformity | 0.0231 | 1.0234 |
| A_log.sigma.5.0.mm.3D_firstorder_Kurtosis | 0.0421 | 1.0430 |
| A_original_ngtdm_Busyness | 0.0868 | 1.0907 |

**Supplementary Table 3**

**Radiomics characteristics in venous phase**

| Features | Coefficient | Exponential coefficient |
| --- | --- | --- |
| V_log.sigma.5.0.mm.3D_glrlm_ShortRunEmphasis | -4.5057 | 0.0110 |
| V_wavelet.HLL_glszm_LowGrayLevelZoneEmphasis | -0.5368 | 0.5846 |
| V_original_glszm_LowGrayLevelZoneEmphasis | -0.3263 | 0.7216 |
| V_logarithm_firstorder_Skewness | -0.0445 | 0.9564 |
| V_logarithm_firstorder_Mean | 0.0001 | 1.0001 |
| V_wavelet.HLL_glszm_GrayLevelNonUniformity | 0.0011 | 1.0011 |
| V_squareroot_firstorder_Maximum | 0.0020 | 1.0020 |
| V_original_shape_Maximum2DDiameterSlice | 0.0036 | 1.0036 |
| V_original_shape_MinorAxisLength | 0.0059 | 1.0059 |
| V_log.sigma.5.0.mm.3D_firstorder_Kurtosis | 0.0174 | 1.0176 |

**Supplementary Table 4**

**Radiomics characteristics of the combined arteriovenous phase**

| Feature | Coefficient | Exponential coefficient |
| --- | --- | --- |
| V_log.sigma.5.0.mm.3D_glrlm_ShortRunEmphasis | -2.7022 | 0.0671 |
| A_original_shape_Sphericity | -0.2110 | 0.8098 |
| V_wavelet.HLL_glszm_LowGrayLevelZoneEmphasis | -0.1997 | 0.8190 |
| A_wavelet.LLH_glcm_Imc2 | -0.1314 | 0.8769 |
| V_logarithm_firstorder_Skewness | -0.0325 | 0.9680 |
| A_logarithm_firstorder_Mean | 0.0004 | 1.0004 |
| V_squareroot_firstorder_Maximum | 0.0008 | 1.0008 |
| V_logarithm_glcm_JointAverage | 0.0021 | 1.0021 |
| V_log.sigma.5.0.mm.3D_firstorder_Kurtosis | 0.0029 | 1.0029 |
| A_original_shape_Maximum2DDiameterColumn | 0.0034 | 1.0034 |
| A_original_shape_Maximum2DDiameterSlice | 0.0045 | 1.0045 |
| A_log.sigma.4.0.mm.3D_glszm_GrayLevelNonUniformity | 0.0172 | 1.0173 |
| A_log.sigma.5.0.mm.3D_firstorder_Kurtosis | 0.0265 | 1.0269 |
| A_original_ngtdm_Busyness | 0.0561 | 1.0577 |

**The R-score calculation equation**

R-score=-(V_log.sigma.5.0.mm.3D_glrlm_ShortRunEmphasis×2.7022) -

(A_original_shape_Sphericity×0.2110) -

(V_wavelet.HLL_glszm_LowGrayLevelZoneEmphasis×0.1997) -

(A_wavelet.LLH_glcm_Imc2×0.1314) -

(V_logarithm_firstorder_Skewness×0.0325)+

(A_logarithm_firstorder_Mean×0.0004)+ (V_squareroot_firstorder_Maximum×0.0008)+

(V_logarithm_glcm_JointAverage×0.0020)+

(V_log.sigma.5.0.mm.3D_firstorder_Kurtosis×0.0028)+

(A_original_shape_Maximum2DDiameterColumn×0.0038)+

(A_original_shape_Maximum2DDiameterSlice×0.0045)+

(V_log.sigma.4.0.mm.3D_glszm_GrayLevelNonUniformity×0.0172)+

(A_log.sigma.5.0.mm.3D_firstorder_Kurtosis×0.0265)+

(A_original_ngtdm_Busyness × 0.0561)
